# Supplementary material for: Complex‐centric proteome profiling by SEC‐SWATH‐MS
Source: Mol Syst Biol. 2019 Jan 14;15(1):e8438. doi: 10.15252/msb.20188438 (PMC6346213; doi:10.15252/msb.20188438)
Supplement: Supplementary file 8 — Dataset EV7 [file MSB-15-e8438-s008.zip › feature_plots_string/P49773.pdf]

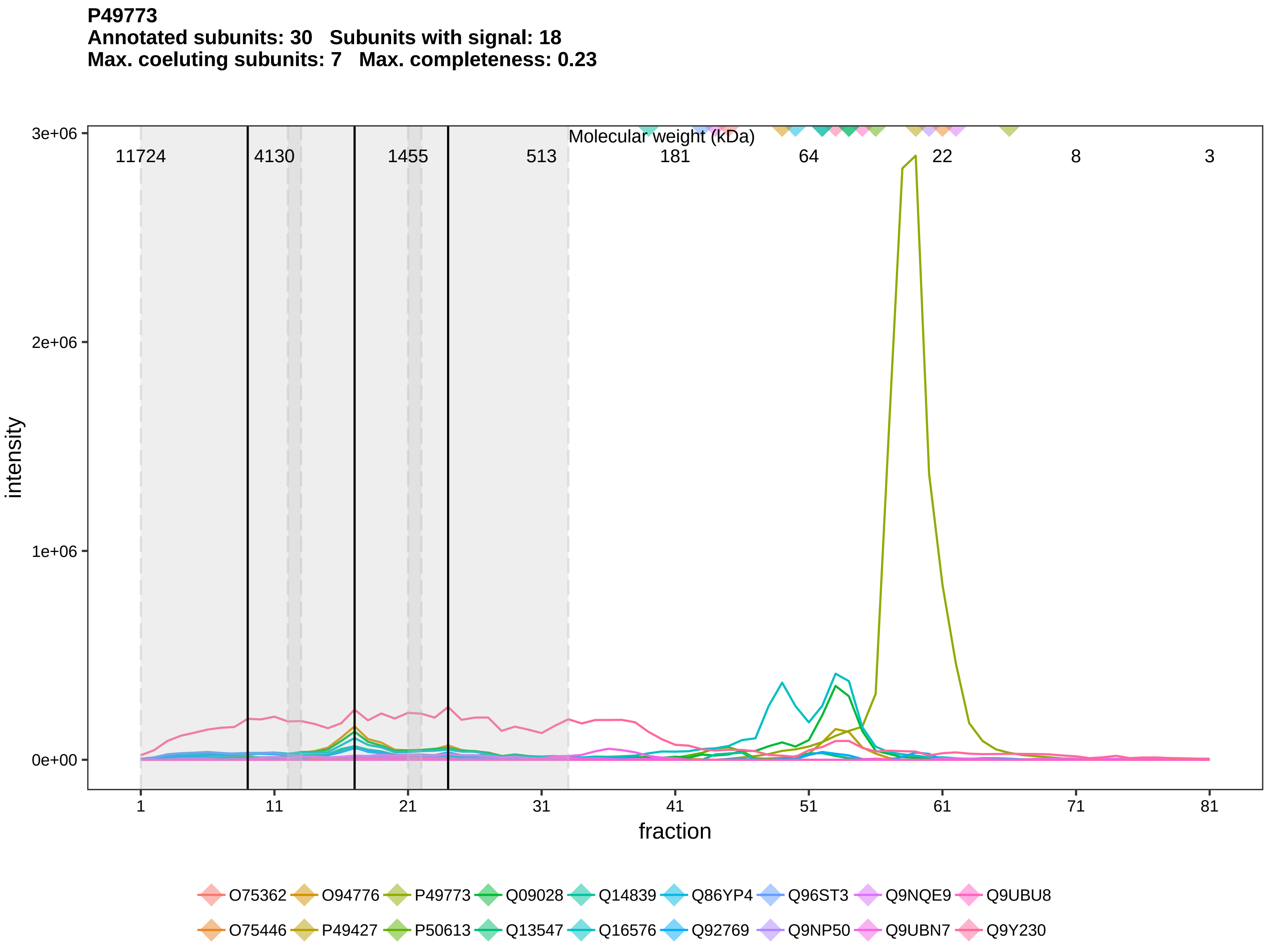
P49773
Annotated subunits: 30   Subunits with signal: 18
Max. coeluting subunits: 7   Max. completeness: 0.23
Molecular weight (kDa)
11724
4130
1455
513
181
64
22
8
3
3e+06
2e+06
1e+06
0e+00
intensity
1
11
21
31
41
51
61
71
81
fraction
O75362
O94776
P49773
Q09028
Q14839
Q86YP4
Q96ST3
Q9NQE9
Q9UBU8
O75446
P49427
P50613
Q13547
Q16576
Q92769
Q9NP50
Q9UBN7
Q9Y230
